# Epidemiology, Genetic Characterization, and Pathogenesis of Avian Influenza H5N8 Viruses Circulating in Northern and Southern Parts of Egypt, 2017–2019

**DOI:** 10.3390/ani11082208

**Published:** 2021-07-26

**Authors:** Mohamed Tarek, Mahmoud M. Naguib, Abdel-Sattar Arafa, Laila A. Tantawy, Karim M. Selim, Shaimaa Talaat, Hesham A. Sultan

**Affiliations:** 1 Reference Laboratory for Veterinary Quality Control on Poultry Production, Animal Health Research Institute, Agriculture Research Center, Giza 12618, Egypt; vet.m.tarek@gmail.com (M.T.); araby85@hotmail.com (A.-S.A.); Dr.kareemselee_87@yahoo.com (K.M.S.); 2 Zoonosis Science Center, Department of Medical Biochemistry and Microbiology, Uppsala University, SE-75237 Uppsala, Sweden; 3 Department of Poultry Disease, Animal Health Research Institute, Agriculture Research Center, Giza 12618, Egypt; rowaina1989@gmail.com; 4 Department of Birds and Rabbits Medicine, University of Sadat City, Sadat 32511, Egypt; shaimaa610@gmail.com

**Keywords:** influenza, poultry, Egypt, phylogenetic relatedness, pathogenicity

## Abstract

**Simple Summary:**

During 2020–2021, highly pathogenic avian influenza (HPAI) viruses of subtype H5N8 were spreading rapidly, and two genetically distinct lineages were detected in Europe, the Middle East, and Southeast Asia. HPAI H5N8 viruses have been circulating in Egyptian poultry flocks since 2016. In this study, 74 commercial chicken farms tested positive for HPAI H5N8 virus. Genetic characterization of the hemagglutinin (HA) and the neuraminidase (NA) of Egyptian HPAI H5N8 viruses showed a relationship with those recently isolated in Europe.

**Abstract:**

Highly pathogenic avian influenza (HPAI) viruses of subtype H5N8 continue to circulate, causing huge economic losses and serious impact on poultry production worldwide. Recently, HPAIV H5N8 has been spreading rapidly, and a large number of HPAI H5N8 outbreaks have been reported in Eurasia 2020–2021. In this study, we conducted an epidemiological survey of HPAI H5N8 virus at different geographical locations in Egypt from 2017 to 2019. This was followed by genetic and pathogenic studies. Our findings highlight the wide spread of HPAI H5N8 viruses in Egypt, including in 22 governorates. The genetic analyses of the hemagglutinin (HA) and neuraminidase (NA) gene segments emphasized a phylogenetic relatedness between the Egyptian HPAI H5N8 viruses and viruses of clade 2.3.4.4b recently isolated in Europe. These findings suggest that a potential back transmission of Egyptian HPAI H5N8 virus has occurred from domestic poultry in Egypt to migratory wild birds, followed by further spread to different countries. This highlights the importance of continuous epidemiological and genetic studies of AIVs at the domestic–wild bird interface.

## 1. Introduction

Outbreaks of highly pathogenic avian influenza (HPAI) viruses among poultry populations have resulted in devastating economic damages to the poultry industry worldwide [1]. In addition, HPAI viruses have shown the ability to cross the species barrier and infect humans and other mammals, posing a serious threat to both human and animal health [2].

In 2010, HPAI H5N8 subtype was reported in China as a result of novel reassortant of hemagglutinin (HA) and neuraminidase (NA) of different AI subtypes [3], which was assigned later within clade 2.3.4.4 [4]. In January 2014, a new reassortant of the HPAI H5N8 virus, clade 2.3.4.4a, was found in both wild and domestic birds in South Korea [5] and further disseminated to other countries in Asia and as far as Europe and North America [6,7]. In 2016/2017, another novel reassortant HPAI H5N8 virus, clade 2.3.4.4b, was reported in Russia and subsequently spread via migratory birds to many countries in Europe, Asia, and Africa within few months causing the most widespread HPAI virus epidemic in the last decade [8,9,10]. Since 2016/2017, HPAI H5N8 viruses of clade 2.3.4.4b have undergone continual evolutionary divergence via reassortment with other influenza A subtypes resulting in various genotypes [8].

In Egypt, the HPAI H5N1 virus has been endemic in poultry populations since 2006 [11]. In December 2016, the HPAI H5N8 virus was first reported in Egypt via migratory birds (common coots (*Fulica atra*)) in the Damietta governorate [12]. In a short time, the virus spread to infect domestic poultry in different geographical locations, causing a great economic loss to local poultry farming [13,14]. The virus became endemic in poultry in Egypt and was found to be the most commonly detected H5 subtype among poultry species [15,16]. The virus was phylogenetically related to viruses of clade 2.3.4.4b reported in Russia 2016 [17]. Genetic and phylogenetic analyses of the Egyptian HPAI H5N8 viruses revealed that at least six different genotypes are circulating in Egypt [13,17,18,19]. In 2018–2019, novel HPAI H5N2 viruses were found in commercial chicken and duck farms in Egypt, as a result of genetic reassortment between HPAI H5N8 and low pathogenic AI (LPAI) H9N2 subtypes circulating in Egypt [20,21]. The HPAI H5N8 viruses reported in the second half of 2020 in Europe were described to be phylogenetically related to HPAI H5N8 viruses isolated in Egypt in 2019 [22,23]. In 2021, this subtype was reported for the first time in humans with a history of contact with infected poultry [24]

Here we study the molecular epidemiology and genetic characterization of the HPAI H5N8 virus in different geographical locations in a temporal manner from 2017 to 2019. In addition, we assess the pathogenicity of two HPAI H5N8 viruses of chicken and duck origin.

## 2. Materials and Methods

### 2.1. Samples Collection

Tracheal swabs were collected from 180 commercial chicken farms showing respiratory signs and mortality. Commercial chicken farms are located in 22 governorates in Upper and Lower Egypt. Samples were submitted to the Reference Laboratory of Veterinary Quality Control on Poultry Production (RLQP), Animal Health Research Institute, Egypt, for virus identification and isolation. Ten tracheal swabs were obtained from each flock and pooled together as one sample. The epidemiological data, mortality rate, and vaccination history of the positive samples were obtained and recorded from all chicken farms as shown in Supplementary Table S1.

### 2.2. Molecular Diagnosis and Virus Isolation

Viral RNA was extracted from the collected samples using the QIAamp Viral RNA Mini Kit (Qiagen, Hilden, Germany) according to the manufacturer’s instructions. All samples were initially tested using standard quantitative reverse transcription polymerase chain reaction (RT-qPCR) specific for the M gene of influenza A viruses [25]. Positive RNAs were further tested using gene-specific RT-qPCR assays for the hemagglutinin (HA) and neuraminidase (NA) gene segments of the AIV H5N8 subtype [26]. Viral RNAs were further tested for the presence of infectious bronchitis virus (IBV) [27] and Newcastle disease virus (NDV) [28]. All RT-qPCR reactions were performed using Stratagene MX3005P real-time PCR machine (Agilent, Santa Clara, CA, USA). Virus isolation was performed through allantoic fluid inoculation of 10-day-old specific-pathogen-free (SPF) embryonated chicken eggs (ECEs) according to the standard protocols of the OIE diagnostic manual [29]. SPF ECEs were obtained from the Nile SPF project (Koom Oshiem, Fayoum, Egypt). The harvested allantoic fluids were tested for virus hemagglutination activity by hemagglutinin assay and verified by using RT-qPCR.

### 2.3. Sequencing and Phylogenetic Analyses

Complete gene segments of the HA and NA were amplified using primers previously described by Hoper et al. [30]. The gene-specific RT-PCR amplicons were size-separated by agarose gel electrophoresis, excised, and purified from gels using the QIAquick Gel Extraction Kit (Qiagen, Hilden, Germany). Further, purified PCR products were used directly for cycle sequencing reactions using BigDye Terminator v3.1 Cycle Sequencing Kit (Applied Biosystems, Waltham, MA, USA). Reaction products were purified using Centrisep spin column (Thermo Fisher, Waltham, MA, USA) and sequenced on an ABI PRISM 3100 Genetic Analyzer (Life Technologies, Carlsbad, CA, USA). Thereafter, the obtained sequences of the HA and NA genes were assembled and edited using the Geneious Prime software, version 2019.1.1 [31]. A Blast search was performed using Global Initiative on Sharing All Influenza Data (GISAID) platform, and sequences established in this study were submitted to GenBank. Additionally, genetic sequences of representative HPAI H5N8 viruses were retrieved from the NCBI and GISAID platforms on 28 November 2020. The nucleotide sequences were edited using BioEdit [32], and alignment analyses were performed using MAFFT [33]. Phylogenetic analysis was performed by employing maximum likelihood methodology based on Akaike criterion after selection of the best fit modes using IQ-tree software version 1.1.3 [34]. Trees were finally viewed and edited using FigTree v1.4.2 software (http://tree.bio.ed.ac.uk/software/figtree/, accessed on 5 March 2021).

### 2.4. Ethical Approval

White Leghorn chickens were hatched from SPF ECEs that were purchased from Nile SPF Farm, Kom Oshiem, Fayom, Egypt, and raised at NLQP. Birds were housed in isolation units, where feed and water were provided daily. All animal experiments in this study were conducted in accordance with guidelines of laboratory animal use and legally approved by the Committee of Ethics of Animal Experiments at the Animal Health Research Institute, Egypt, under protocol number (AHRI-2429). Infection experiments were performed in isolators at animal biosafety level 3 (BSL-3).

### 2.5. Animal Experiment “Intravenous Pathogenicity Index (IVPI)”

Intravenous pathogenicity index (IVPI) was assessed using the virus isolated from this study (A/chicken/Egypt/S30/2019 (chicken/S30)) and compared to another recent HPAI H5N8 (A/duck/Egypt/SMG4/2019 (duck/SMG4)) virus. The chicken/S30 was isolated from a broiler chicken farm (31 days old) in February 2019 in Sinai governorate (GenBank accession No. MN658696). The farm was not vaccinated against HPAI H5 virus, and the mortality rate was 33.5%. The latter virus, duck/SMG4, was obtained from our virus repository. The virus was previously isolated from a Muscovy duck farm in January 2019 in Port Said governorate (GenBank accession No. MN658766). The farm was also not vaccinated against HPAI H5 virus, and the mortality rate was 23%. Two groups of 10 chickens each were inoculated intravenously with 0.1 mL of positive allantoic fluid containing 10^6^ EID_50_/mL of each virus. A negative control group (*n* = 10) was inoculated with phosphate-buffered saline (PBS). Chickens were monitored daily for clinical signs and mortality 10 days post-infection (dpi). The IVPIs were determined according to a standardized clinical scoring system of the Office International des Epizooties (OIE) [35]. Clinical signs were scored as follows: 0 = healthy (no signs); 1 = sick (showing one of the following symptoms: ruffled feathers, respiratory manifestations, depression, facial edema, cyanosis of comb and wattles, or diarrhea); 2 = dead. Survival rates were observed for 10 days following challenge with HPAI H5N8 virus. Survival curve was generated based on Kaplan–Meier survival curves and visualized using survival and ggplot2 packages in R Version 4.0.2 [36,37]. Further, *p*-value for comparison between survival curves was calculated using log-rank test (argument rho = 0 while using survfit function survival package V3.2.11). Individual oropharyngeal and cloacal swabs were collected at dpi 2 from chickens and tested for influenza virus M gene by RT-qPCR as described in the previous section. Dead chickens from each group were dissected, and organs (trachea, spleen, liver, pancreas, cerebrum, cecal tonsil, and bursa of Fabricius) were collected for histopathological examination. Examined tissues were preserved in 10% buffered formalin. Formalin-fixed paraffin-embedded tissues were processed, sectioned, and stained with H&E [38]. Ordinal scoring system of lesions of infected tissues was applied according to the progression of the severity; lesions were scored as − (normal), + (mild), ++ (moderate), or +++ (severe) as previously described by [39]. Five random optical fields were examined and scored, and then the mean of the five fields was calculated.

## 3. Results

### 3.1. Geographical Distribution and Seasonal Spread of HPAI H5N8 Viruses in Egypt

**Figure 1:**
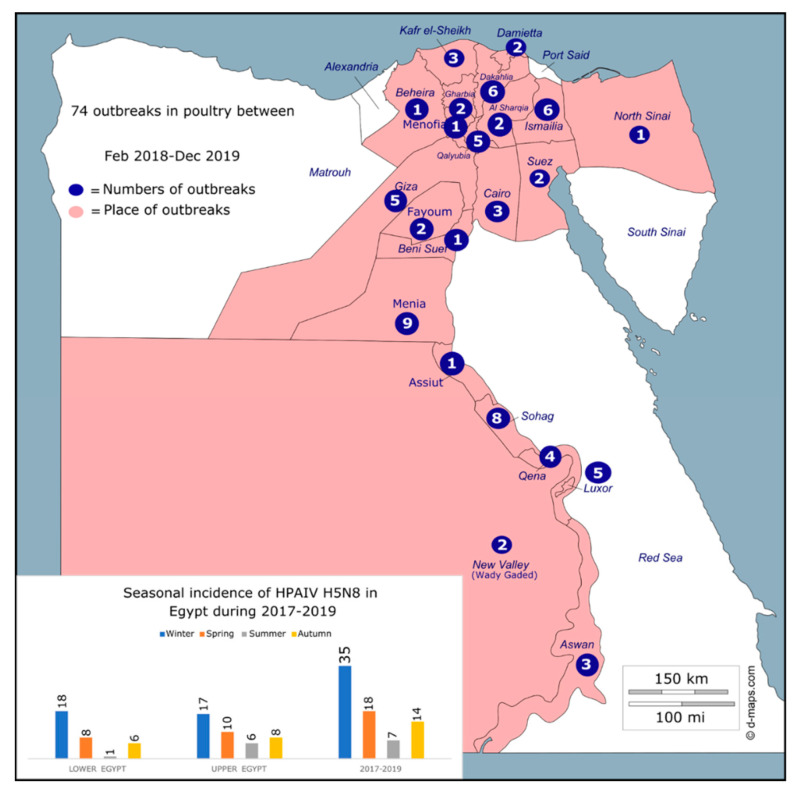
Temporal (graph) and geographic (map) distribution of outbreaks of HPAI H5N8 virus in poultry in Egypt, 2017–2019. Map of Egypt is modified from https://d-maps.com/carte.php?num_car=25356&lang=en, accessed on 21 July 2021.

All collected samples (total number of 74 commercial chicken farms) were found to be positive for the HPAI H5N8 virus in 22 governorates in Egypt (Figure 1 and Supplementary Table S1). The Cq value obtained from the RT-qPCR of each sample is shown in Supplementary Table S1. Mortality rates were ranged from 5% in vaccinated farms to 43.1% in nonvaccinated farms (Supplementary Table S1). Virus isolation was successful from 11 samples (Supplementary Table S2). An increased number of positive cases of HPAI H5N8 was found in broiler chicken farms compared to layer farms (52 broiler vs. 22 layer) (Supplementary Table S1).

To better analyze the geographical distribution of HPAI H5N8 viruses Egypt, the 22 governorates were divided into two different regions on the basis of geographic location, Upper and Lower Egypt. A total of 40 positive farms were detected in Upper Egypt, and 34 were detected in Lower Egypt (Figure 1, Supplementary Table S1). The highest number of detections of the HPAI H5N8 virus was in the Menia governorate (*n* = 9). Remarkably, outbreaks were strongly associated with the winter season, in which 35 out of 74 positive cases were found, and substantially decreased during the summer season, in which only 7 positive cases were found (Figure 1).

### 3.2. Genetic and Phylogenetic Characterization

Sequences were generated for HA and NA gene segments of 11 Egyptian H5N8 isolates representing years 2017 (*n* = 4), 2018 (*n* = 3), and 2019 (*n* = 4) (Supplementary Table S2). The obtained sequences were submitted to GenBank under the accession number shown in Supplementary Table S2. The nucleotide homology analysis showed a similarity range of 95–99% among the 11 H5N8 viruses sequenced in this study for both the HA and the NA.

Molecular analysis revealed that the HA cleavage site of the Egyptian HPAI H5N8 virus had a multiple basic amino acid motif “PLREKRRKR/GLF” in all viruses sequenced in this study (Supplementary Table S2) except A/chicken/Egypt/AL1/2019 with “PIREKRRKR/GLF” motif; both motifs presented HPAI properties. The Q226L or G228S amino acid mutations in the HA, which are essential for adaptation of avian HA to mammals [40], were not found in the H5N8 viruses in this study. Further, no deletions in the stalk region of the NA protein were recorded among the 11 sequenced Egyptian HPAI H5N8 viruses of this study.

**Figure 2:**
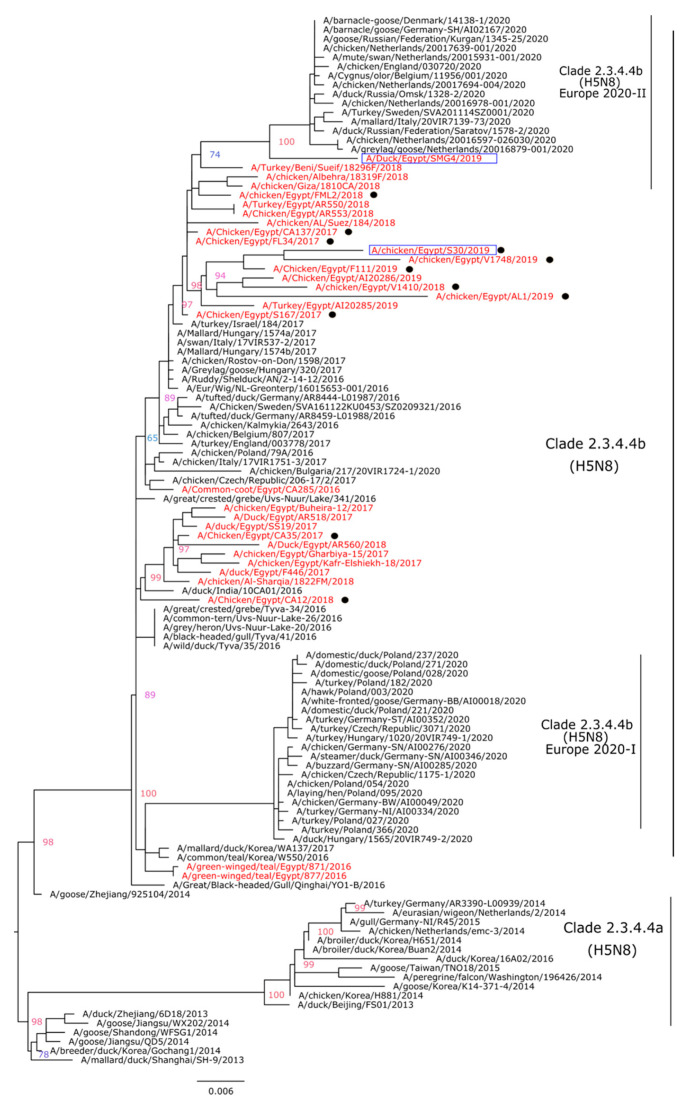
Phylogenetic tree of the HA gene segment of HPAI H5N8 viruses. A phylogenetic tree including a total of 114 HA segments from different H5N8 viruses was obtained using the IQTree software after selection of best fit model (K3Pu + F + G4). Egyptian HPAI H5N8 viruses are colored in red; viruses of the current study (*n* = 11) are indicated with black dots. The two Egyptian HPAI H5N8 viruses selected for IVPI are shown in blue boxes.

**Figure 3:**
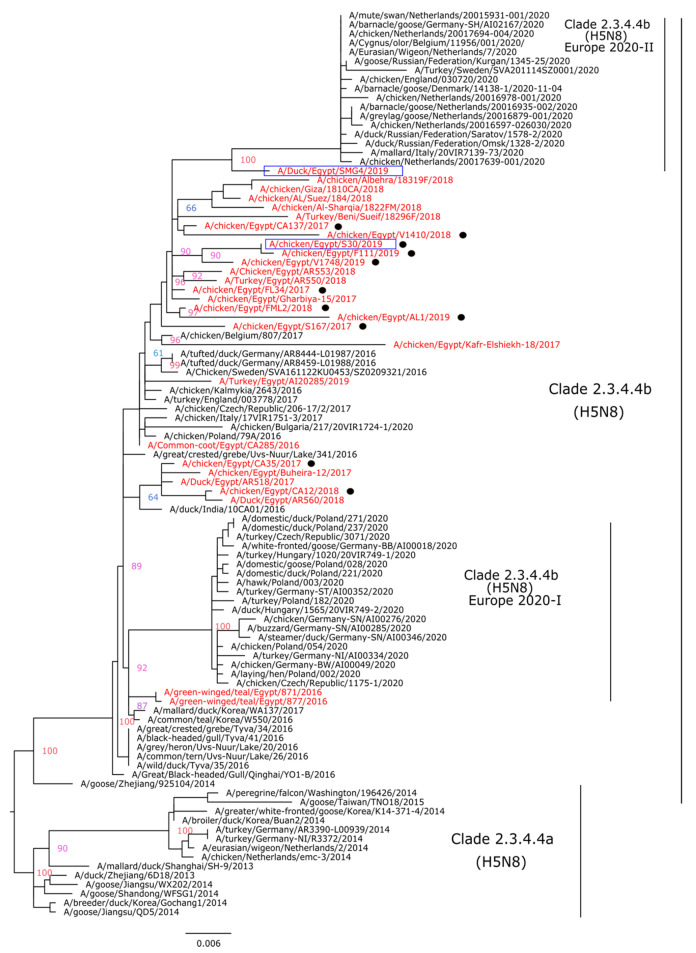
Phylogenetic tree of the NA gene segment of HPAI H5N8 viruses. A phylogenetic tree including a total of 99 NA segments from different H5N8 viruses was obtained using the IQTree software after selection of best fit model (K3Pu + F + G4). Egyptian HPAI H5N8 viruses are colored in red; viruses of the current study (*n* = 11) are indicated with black dots. The two Egyptian HPAI H5N8 viruses selected for IVPI are in blue boxes.

Phylogenetic analysis indicated that the HA genes of the Egyptian HPAI H5N8 viruses isolated in this study are clustered with other H5N8 viruses of clade 2.3.4.4.b (H5N8) reported in 2016–2018 and could be the possible progenitors of the HPAI H5N8 viruses identified for the first time in Eurasia in July 2020 (Figure 2). Indeed, the N8 gene phylogenetic tree revealed the same pattern as seen in HA and indicated that the studied HPAI H5N8 viruses are grouped with N8 genes of viruses isolated previously in Egypt and recent HPAI H5N8 viruses in Eurasia (Figure 3).

### 3.3. Pathogenesis of HPAI H5N8 Viruses of Chicken and Duck Origin

We compared the IVPI of chicken/S30 and duck/SMG4 isolated in 2019 from two bird species. The HA and NA genes of the duck/SMG4 are shown to be a putative ancestor for the recent HPAI H5N8 viruses circulating in Europe (Figure 2 and Figure 3). After intravenous infection with A/chicken/Egypt/S30/2019, all 10 inoculated chickens died within 3 days, resulting in an intravenous pathogenicity index (IVPI) of 2.83; while with the duck/SMG4, only 9 chickens died, resulting in an IVPI of 2.41. No symptoms were observed in the negative control group.

**Figure 4:**
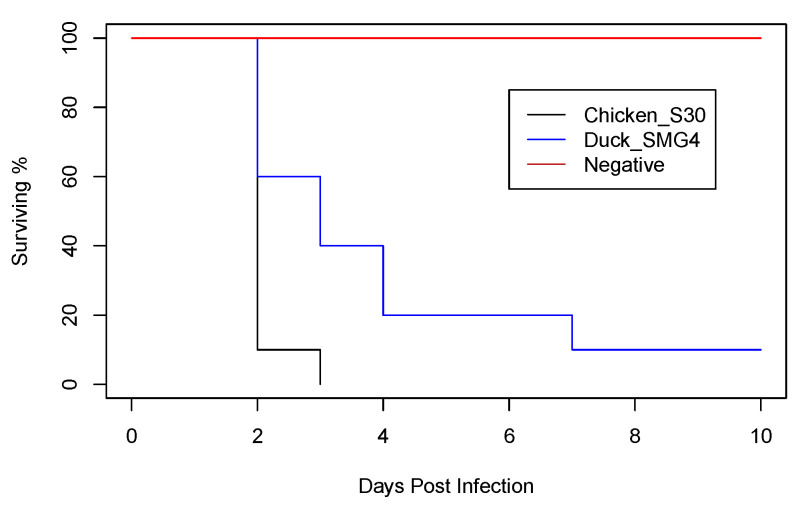
Survival rate showing the daily mortality in each chicken group after infection.

A virus titer of 1.95 × 10^5^ was detected at 2 dpi in the chicken/S30-infected group, whereas that in the duck/SMG4-infected group was 9.18 × 10^4^ (Table 1). Notably, prolonged virus shedding, up to 10 dpi, was observed in the duck/SMG4-infected group (Table 1). The chicken/S30 caused deaths of all infected chickens within 48–72 h (Figure 4), while duck/SMG4 produced mortality of only 90% of infected chickens. A statistically significant difference between the groups was found, where the resulting *p*-value for the log-rank test was <0.00001.

Post mortem inspection of gross lesions of infected chickens revealed similar lesions, including multifocal petechiae and necrotic areas in the pancreas, congestion in the central nervous system, lung consolidation, diffuse congestion in internal organs, and multifocal petechiae in the bursa of Fabricius. No macroscopic lesions were observed in negative control birds.

Microscopic examination of the tissues collected from dead or severely affected chickens revealed evident lesions of variable intensity in mostly all the collected organs. However, we detected differences in the severity and viral replication in the different tissues between chicken/S30 and duck/SMG4. The mean severity index was 2.42 for A/chicken/Egypt/S30/2019 and 1.75 for duck/SMG4.

**Figure 5:**
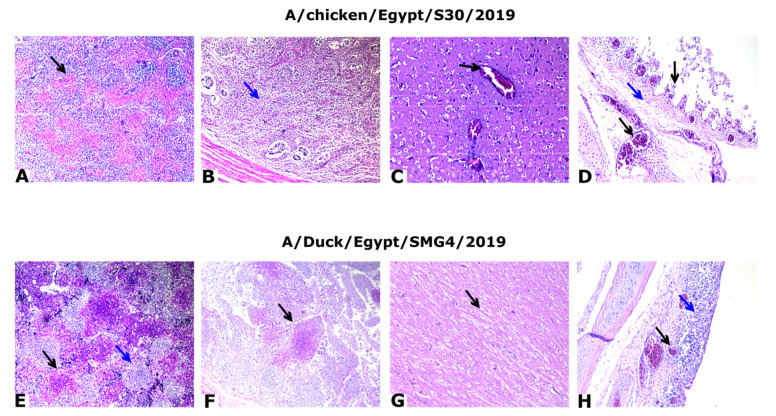
Histopathology slices from the tissue of infected chickens. (**A**) Spleen of A/chicken/Egypt/S30/2019(H5N8) at 2 dpi showing severe multifocal necrotic tissue (black arrow); H&E, ×100. (**B**) Cecal tonsils at 2 dpi showing mild depletion of lymphocytes (star); H&E, ×100. (**C**) Cerebrum of control positive at 3 dpi showing congested blood vessels (black arrow); H&E, ×200. (**D**) Trachea of control positive at 2 dpi showing sloughed epithelium, edema (blue arrow), and congested blood vessels in lamina propria (black arrow); H&E, ×200. (**E**) Spleen of A/Duck/Egypt/SMG4/2019(H5N8) at 2 dpi showing severe depletion of lymphocytes (blue arrow) with multifocal necrotic areas (black arrow); H&E, ×100. (**F**) Cecal tonsils at 2 dpi showing multifocal necrotic patches (black arrow); H&E, ×100. (**G**) Cerebrum at 2 dpi showing demyelination (black arrow); ×200. (**H**) Trachea at 2 dpi showing thickening of the wall of the mucosal layer due to congested blood vessels (black arrow), edema, and mononuclear cell infiltration (blue arrow); H&E, ×200.

The most relevant microscopic lesions were observed in the spleen, cecal tonsils and cerebrum. The lesions were more severe in the case of chicken/S30 isolate than in the case of the duck/SMG4 isolate. Spleens showed depletion of lymphocytes (Figure 5A,E), in addition to multifocal necrosis of splenocytes and congested blood vessels. Cecal tonsils showed mild to severe depletion of lymphocytes and necrosis (Figure 5B,F). Further, the cerebrum showed degeneration of neurons associated with perineural edema, as well as central chromatolysis associated with demyelination and perineural edema (Figure 5C,G). The trachea exhibited loss of lining epithelium with edema, congestion, and mononuclear cell infiltration in the lamina propria in the case of the duck/SMG4 isolate (Figure 5H). Detailed organ scoring is shown in Supplementary Table S3. All organs in negative control birds showed apparently normal structures.

## 4. Discussion

Influenza A (H5N8) viruses have been the dominant H5 subtype circulating among bird species since 2014 [8]. The aim of the present study was to determine (i) the geographical spread of HPAI H5N8 virus in chicken farms in Egypt in 2017–2019, (ii) the genetic and phylogenetic features of the recent HPAI H5N8 viruses circulating in Egypt, and (iii) the intravenous pathogenicity index of two HPAI H5N8 viruses isolated from different species (chickens vs. duck). During 2020–2021, HPAI H5N8 viruses were frequently isolated from wild birds and domestic poultry in several countries worldwide [23,41]. Recently, the HPAI H5N8 virus was detected for the first time in humans working at an infected chicken farm in Russia [24].

In total, 74 chicken farms were found positive for the HPAI H5N8 virus in 22 governorates in Lower and Upper Egypt, suggesting a wide spread and continuous circulation of this virus after its incursion in 2016. The numbers of HPAI H5N8 outbreaks can vary based on different factors including poultry density and climate, where cold temperature and low humidity have been shown to favor virus spread [42,43]. The results of this study demonstrated that is a relationship between climate factors and the number of outbreaks; for example, most HPAI H5N8 cases were found in the winter season. Further, in Egypt, several influenza A subtypes are co-circulating among poultry species, including HPAI viruses H5N1, H5N2, and H5N8 and the LPAI H9N2 virus [20,21]. The wide detection of HPAI H5N8 viruses in this study confirms previous findings of H5N8 spread in Egypt [13,17]. This must be considered as this increases the chances of reassortment and emergence of new subtypes/genotypes. Further, the Egyptian HPAI H5N8 viruses of this study showed a high genetic similarity at the level of HA and NA gene sequence with viruses isolated recently from Europe and Korea in both wild and domestic birds. In addition, phylogenetic analyses demonstrated that HPAI H5N8 viruses detected in Egypt are closely related to those viruses, suggesting that the Egyptian HPAI H5N8 viruses are potential progenitors of the recent HPAI H5N8 viruses identified in Europe and Southeast Asia. These findings can suggest that a potential back transmission of the Egyptian HPAI H5N8 virus has occurred from domestic poultry in Egypt to migratory wild birds, followed by further spread to different countries. To confirm this hypothesis, the investigation of samples collected in early 2020 and the collection of new samples from locations where wild birds interact with domestic poultry are required. Further, the Egyptian HPAI H5N8 virus that was isolated from domestic chickens (chicken/S30) demonstrated significantly higher mortality and IVPI compared to the virus isolated from ducks (duck/SMG49). However, both viruses revealed an IVPI of >1.2, which indicates high pathogenicity. Understanding the mechanisms behind this variation is a matter of interest, and further study is required to explore the transmission and the pathobiology of the two viruses via the natural route of infection.

In the sense of the recent human cases of the HPAI H5N8 virus in Russia, we recommend that all poultry farmers in Egypt should be vigilant and be aware of the current biosafety and biosecurity recommendations. Early warning and rapid reporting of new cases might minimize the risk of virus spread and detection of new cases. The stamping-out policy, typically implemented by culling infected and suspected poultry in Egypt, should be reevaluated and implemented in a more efficient way. Continuous surveillance and whole genomic sequencing of the virus at the domestic–wild bird interface is essential to understand the evolution of this subtype and the intercontinental transmission dynamics of the HPAI H5N8 virus.

## Figures and Tables

**Table 1:** Virus detection in infected groups of chickens. Table presents mean values detected from swab samples collected from inoculated chickens.

Day pi	A/Chicken/Egypt/S30/2019(H5N8)		A/Duck/Egypt/SMG4/2019(H5N8)	
	CT	Titer (EID50/mL)	CT	Titer (EID50/mL)
D2	17.41	1.95 × 10^5^	19.22	9.18 × 10^4^
D3	18.51	8.53 × 10^4^	18.45	1.84 × 10^5^
D6	dead	dead	20.36	1.59 × 10^4^
D10	dead	dead	29.31	2.13 × 10^2^

## Data Availability

The obtained sequences in this study were submitted to GenBank under the accession number shown in Supplementary Table S2.

## Supplementary Materials

The following are available online at https://www.mdpi.com/article/10.3390/ani11082208/s1: Table S1. Epidemiological data and RT-qPCRa results of positive HPAI H5N8 viruses in this study. All sequenced viruses (n = 11) were found within clade 2.3.4.4b as shown in Figure 2 and Figure 4. Table S2. Haemagglutinin Cleavage site and accession number of HA and NA genes. Table S3. Scoring lesions of infected tissues.
