# Supplementary material for: Epidemiology, Genetic Characterization, and Pathogenesis of Avian Influenza H5N8 Viruses Circulating in Northern and Southern Parts of Egypt, 2017–2019
Source: Animals (Basel). 2021 Jul 26;11(8):2208. doi: 10.3390/ani11082208 (PMC8388380; doi:10.3390/ani11082208)
Supplement: Supplementary file 1 [file animals-11-02208-s001.zip › animals-1258865-supplementary.pdf]

animals MDPI

Supplementary Tables and Figures

Supplementary Table S1. Epidemiological data and RT-qPCR[a] results of positive HPAI H5N8 viruses in this study. All sequenced viruses (n = 11) were found within clade 2.3.4.4b as shown in Figures 2 and 4.

No.	Date	Governorate	Age/days	Bird type	Mortality%	Vaccination regime		RT-qPCR/Cq[a]
						Frequency	H5-clade	
1	Jan. 2017	Sharkia	23	Broilers	12.5%	1X	2.2.1	18
2	Jan 2017	Qalyubia	18	Broilers	8.5%	1X	2.2.1	15
3*	Jan 2017	Suez	26	Broilers	23.5%	1X	2.2.1	29
4	Jan 2017	Cairo	34	Broilers	25.33%	1X	2.2.1	26
5	Feb 2017	Dakahlia	33	Broilers	19.42%	1X	2.2.1	22
6	Feb 2017	Ismailia	24	Broilers	35%	1X	2.3.2	22
7	Feb 2017	Qena	26	Broilers	10.42%	1X	2.2.1	24
8	Feb 2017	Kafer El-Sheikh	70	layers	11.2%	2X	2.3.4	12
9	Feb 2017	Sohag	31	Broilers	15%	1X	2.2.1	18
10	Mar 2017	Sohag	23	Broilers	19.2%	1X	2.3.2	21
11	Mar 2017	Sohag	29	Broilers	15.08%	1X	2.3.2	23
12	Mar 2017	Luxor	22	Broilers	17%	non-vaccinated	non-vaccinated	20
13	Mar 2017	Sohag	18	Broilers	16%	1X	2.3.2	16
14	Mar 2017	Qalyubia	26	Broilers	23.8%	non-vaccinated	non-vaccinated	19
15	Mar 2017	Sohag	25	Broilers	16.8%	1X	2.2.1	21
16	Mar 2017	Aswan	31	Broilers	28%	non-vaccinated	non-vaccinated	22
17	Mar 2017	Cairo	33	Broilers	16%	1X	2.3.2	29
18	Mar 2017	Ismailia	52	layers	12%	2X	2.2.1	23
19	May 2017	Luxor	32	Broilers	7.8%	1X	2.2.1	24
20	Jun 2017	Menia	23	Broilers	36.5%	1X	2.2.1	18
21	Jun 2017	Menia	25	Broilers	12.5%	1X	2.2.1	15
22	Jun 2017	Menia	17	Broilers	23.5%	1X	2.3.2	30
23	Jul 2017	Giza	115	layers	11%	3X	2.3.4	26
24	Oct 2017	Menia	31	Broilers	25.33%	1X	2.2.1	14
25	Nov 2017	Sohag	27	Broilers	19.42%	1X	2.2.1	16
26*	Nov 2017	Kafer El-Sheikh	45	layers	8%	1X	2.3.4	24
27	Nov 2017	Ismailia	29	Broilers	31%	1X	2.2.1	12
28	Nov 2017	Giza	78	layers	15%	non-vaccinated	non-vaccinated	18
29	Dec 2017	Qena	28	Broilers	10.42%	1X	2.3.2	21
30*	Dec 2017	Luxor	40	layers	24.2%	non-vaccinated	non-vaccinated	23
31	Dec 2017	Qena	33	Broilers	12.5%	1X	2.3.2	20
32*	Dec 2017	Giza	25	Broilers	30%	1X	2.3.2	16
33	Dec 2017	Menoufia	19	Broilers	19.7%	1X	2.2.1	13
34	Dec 2017	Sohag	15	Broilers	26%	non-vaccinated	non-vaccinated	15
35	Jan 2018	Menia	28	Broilers	14%	1X	2.2.1	18

36	Jan 2018	Suez	23	Broilers	11.2%	1X	2.2.1	23
37	Jan 2018	Wadi El-Gadid	112	layers	7%	3X	2.2.1	24
38	Jan 2018	Sharkia	50	layers	20%	2X	2.3.4	27
39	Feb 2018	Luxor	55	layers	14%	1X	2.3.4	20
40	Feb 2018	Menia	43	layers	12%	1X	2.2.1	21
41	Feb 2018	Giza	32	Broilers	33%	non-vaccinated	non-vaccinated	18
42*	Mar 2018	Giza	123	layer	6.5%	3X	2.3.4	22
43*	Mar 2018	Luxor	20	Broilers	17%	1X	2.3.2	15
44	Mar 2018	Beheira	90	layers	7%	2X	2.2.1	23
45	May 2018	Menia	33	Broilers	20%	1X	2.2.1	21
46	Aug 2018	Kafer El-Sheikh	35	Broilers	30%	non-vaccinated	non-vaccinated	26
47	Dec 2018	Dakahlia	28	Broilers	9%	1X	2.2.1	14
48*	Dec 2018	Qalyubia	35	Broilers	14%	1X	2.3.2	16
49	Dec 2018	Damietta	33	Broilers	11%	1X	2.2.1	22
50	Jan 2019	Gharbia	240	layers	7.8%	4X	2.3.4	19
51*	Feb 2019	Aswan	26	Broilers	16.5%	non-vaccinated	non-vaccinated	20
52	Feb 2019	Wadi El-Gadid	30	Broilers	12.5%	1X	2.2.1	16
53*	Feb 2019	Sinai	31	Broilers	33.5%	non-vaccinated	non-vaccinated	12
54*	Feb 2019	Qalyubia	275	layers	9%	4X	2.3.4	23
55*	Mar 2019	Dakahlia	60	layers	2.33%	2X	2.3.4	18
56	Mar 2019	Cairo	165	layers	19. 2%	3X	2.2.1	12
57	May 2019	Damietta	24	Broilers	12.2%	non-vaccinated	non-vaccinated	23
58	May 2019	Menia	26	Broilers	15%	1X	2.2.1	15
59	Jun 2019	Sohag	30	Broilers	19.2%	1X	2.2.1	28
60	Jun 2019	Beni-Suef	102	layer	19.08%	2X	2.2.1	26
61	Sep 2019	Ismailia	16	Broiler	13%	1X	2.3.2	14
62	Sep 2019	Dakahlia	115	layer	11%	3X	2.3.4	16
63	Oct 2019	Aswan	31	Broiler	43.1%	non-vaccinated	non-vaccinated	24
64	Oct 2019	Assiut	27	Broiler	16.8%	1X	2.2.1	15
65	Nov 2019	Menia	145	layer	9%	2X	2.2.1	29
66	Nov 2019	Qalyubia	29	Broiler	21%	non-vaccinated	non-vaccinated	26
67	Nov 2019	Dakahlia	78	layer	19.2%	1X	2.2.1	22
68	Nov 2019	Dakahlia	23	Broiler	11.5%	1X	2.3.2	22
69	Nov 2019	Ismailia	38	Broiler	18.5%	non-vaccinated	non-vaccinated	22
70	Dec 2019	Qena	93	layer	5%	2X	2.2.1	13
71	Dec 2019	Ismailia	18	Broiler	32.5%	non-vaccinated	non-vaccinated	18
72	Dec 2019	Fayoum	43	Broiler	13%	1X	2.3.2	19
73	Dec 2019	Fayoum	30	Broiler	35%	non-vaccinated	non-vaccinated	23
74	Dec 2019	Gharbia	60	layer	16%	non-vaccinated	non-vaccinated	15

a=Reverse transcription quantitative Polymerase Chain Reaction (Considered positive less than 31 CT). **X** = Times. *Selectedsamples to be isolated

Supplementary Table S2. Haemagglutinin Cleavage site and accession number of HA and NA genes.

Code no.	Designation	Cleavage site of HA protein	HA GenBank Accession no.	NA GenBank Accession no.
3	A/chicken/Egypt/CA35/2017(H5N8)	PLREKRRKR/GLF	MN658687	MN862358
26	A/chicken/Egypt/CA137/2017(H5N8)	PLREKRRKR/GLF	MN658688	MN862359
48	A/chicken/Egypt/V1410/2018(H5N8)	PLREKRRKR/GLF	MN658693	MN862363
53	A/chicken/Egypt/S30/2019(H5N8)	PLREKRRKR/GLF	MN658696	MN862368
54	A/chicken/Egypt/V1748/2019(H5N8)	PLREKRRKR/GLF	MN658697	MN862365
55	A/chicken/Egypt/F111/2019(H5N8)	PLREKRRKR/GLF	MN658695	MN862367
30	A/chicken/Egypt/FL34/2017(H5N8)	PLREKRRKR/GLF	MN658689	MN862360
32	A/chicken/Egypt/S167/2017(H5N8)	PLREKRRKR/GLF	MN658690	MN862361
42	A/chicken/Egypt/CA12/2018(H5N8)	PLRERRRKR/GLF	MN658691	MN862362
43	A/chicken/Egypt/FML2/2018(H5N8)	PLREKRRKR/GLF	MN658692	MN862364
51	A/chicken/Egypt/AL1/2019(H5N8)	PIREKRRKR/GLF	MN658694	MN862366

no.: number

Supplementary Table S3. Scoring lesions of infected tissues

	Day/organ	Trachea	Spleen	Cerebrum	Cecal tonsil
A/duck/Egypt/SMG4/2019	2	++	+++	++	+
	3	+	+++	++	+
	4	+	+++	+	+
	7	++	+	+	++
A/chicken/Egypt/S30/2019	2	++	+++	++	+++
	3	++	+++	++	+++

+:mild ++:moderate +++:Severe
